# A Novel Plant-Based Nutraceutical Combined with Exercise Can Revert Oxidative Status in Plasma and Liver in a Diet-Induced-Obesity Animal Model

**DOI:** 10.3390/antiox13030274

**Published:** 2024-02-23

**Authors:** Ana Guzmán-Carrasco, Garyfallia Kapravelou, María López-Jurado, Francisco Bermúdez, Eduardo Andrés-León, Laura C. Terrón-Camero, José Prados, Consolación Melguizo, Jesus M. Porres, Rosario Martínez

**Affiliations:** 1 Department of Physiology, Institute of Nutrition and Food Technology (INyTA), Biomedical Research Center (CIBM), Sport and Health University Research Institute (IMUDS), Universidad de Granada, 18016 Granada, Spain; anaguzman@correo.ugr.es (A.G.-C.); kapravelou@ugr.es (G.K.); mlopezj@ugr.es (M.L.-J.); rosariomz@ugr.es (R.M.); 2 Cellbitec S.L., N.I.F. B04847216, Scientific Headquarters of the Almería Technology Park, Universidad de Almería, 04128 Almería, Spain; francisco.bermudez@beyond-seeds.com; 3 Department of Anatomy and Embryology, Faculty of Medicine, Institute of Biopathology and Regenerative Medicine (IBIMER), Biomedical Research (CIBM), Instituto Biosanitario de Granada (ibs.GRANADA), University of Granada, 18016 Granada, Spain; jcprados@ugr.es (J.P.); melguizo@ugr.es (C.M.); 4 Bioinformatics Unit, Institute of Parasitology and Biomedicine “López-Neyra” (IPBLN), Consejo Superior de Investigaciones Científicas (CSIC), 18016 Granada, Spain; eduardo.andres@csic.es (E.A.-L.); laura.terron@csic.es (L.C.T.-C.)

**Keywords:** obesity, oxidative stress, natural extracts, antioxidant potential, nutraceutical, physical exercise

## Abstract

The prevalence of obesity increases alarmingly every year mostly due to external factors such as high-fat and high-refined sugar intake associated with a sedentary lifestyle. It triggers metabolic disorders such as insulin resistance, hyperlipemia, non-alcoholic fatty liver disease, chronic inflammation, oxidative stress, and gut microbiota dysbiosis. The aim of this study was to evaluate the beneficial effects of a combined intervention with caloric restriction, nutraceutical intake, and a mixed training protocol on oxidative stress, inflammation, and gut dysbiosis derived from the development of obesity in a C57BL6/J mouse experimental model of diet-induced obesity (4.6 Kcal/g diet, 45% Kcal as fat, and 20% fructose in the drinking fluid). The nutraceutical was formulated with ethanolic extracts of *Argania spinosa* pulp (10%) and *Camelina sativa* seeds (10%) and with protein hydrolysates from *Psoralea corylifolia* seeds (40%) and *Spirodela polyrhiza* whole plants (40%). The combination of nutraceutical and exercise decreased the animals’ body weights and inflammatory markers (TNFα, IL-6, and resistin) in plasma, while increasing gene expression of *cat*, *sod2*, *gsta2*, and *nqo1* in the liver. Obese animals showed lower β-diversity of microbiota and a higher Firmicutes/Bacteroidetes ratio vs. normocaloric controls that were reversed by all interventions implemented. Dietary inclusion of a nutraceutical with high antioxidant potential combined with an exercise protocol can be beneficial for bodyweight control and improvement of metabolic status in patients undergoing obesity treatment.

## 1. Introduction

Obesity is defined as excessive and abnormal fat accumulation mainly associated with an energy imbalance between calorie intake and expenditure. According to the World Health Organization (WHO), obesity is considered an epidemic problem given that its prevalence has tripled worldwide since 1975 [1]. Its incidence increases alarmingly every year mostly due to external factors such as high-fat and high-refined sugar intake, as well as a sedentary lifestyle. Nevertheless, genetic factors are also involved [2]. Obesity triggers metabolic disorders such as insulin resistance or hyperlipemia, which increase the risk of developing non-alcoholic fatty liver disease (NAFLD), chronic inflammation, oxidative stress, and gut microbiota dysbiosis [3]. It is also linked to mitochondrial dysfunction by an increase in reactive oxygen species (ROS) due to higher oxidation caused by an elevated level of free fatty acids. This, in turn, can lead to mitochondrial apoptosis, enhanced lipid accumulation and, consequently, adipocyte hypertrophy. The former leads to a significant inflammatory response, including the secretion of pro-inflammatory cytokines that attract pro-inflammatory macrophages [4]. Therefore, the onset and progression as well as the treatment and prevention of obesity share some common signaling pathways based on glucose and lipid metabolism as well as antioxidant and inflammation response [5]. 

Lifestyle changes such as healthy dietary patterns and daily physical activity are so far the most effective nonsurgical methods to achieve effective weight loss [6]. These strategies modulate the main mechanisms involved in appetite control, restriction of intestinal lipid absorption, inhibition of adipogenesis, lipid mobilization, and microbiota modulation among others [3]. Caloric restriction has been reported to not only normalize body weight in diet-induced obesity models but to also improve different obesity-associated metabolic disorders [7,8]. Moreover, several drugs such as orlistat, setmelanotide, naltrexone/bupropion, liraglutide, and semaglutide are being used as anti-obesity drugs. Specifically, orlistat inhibits gastric and pancreatic lipases and reduces fat absorption by 30%. Liraglutide and semaglutide are glucagon-like peptide-1 (GPL1) receptor agonists that regulate food intake, satiety, and glucose homeostasis. However, the use of these drugs has often been associated with several side effects [4]. In this context, plant-derived bioactive compounds are becoming increasingly relevant in the treatment of obesity and metabolic syndrome. Polyphenols, flavonoids, carotenes, catechins, and isoflavones, among other compounds, promote weight loss and improve health status by neutralizing intracellular ROS that are increased in obese subjects. Furthermore, they can suppress pre-adipocyte differentiation, reduce lipogenesis, increase energy expenditure, and modulate inflammation [6,9]. Thus, the development of new plant-derived nutraceutical formulas with anti-obesity action and no adverse effects on human health represents an innovative strategy for reducing obesity and its related disorders.

The gut microbiota is also accepted as one of the main environmental factors involved in the development of obesity [10]. Moreover, the high capacity to absorb energy by intestinal microbiota is essential in regulating fat accumulation, energy homeostasis, and mucosal barrier integrity. Obesity is frequently associated with an imbalance in the gut bacterial community and a lower diversity, both of which affect its functionality [11]. An abnormal lifestyle leads to intestinal changes associated with adiposity such as a higher Firmicutes/Bacteroidetes (F/B) ratio and an increase in intestinal pH. Such alterations may cause a loosening of luminal junctions and lipopolysaccharide leakage that leads to insulin resistance. Different bioactive compounds from plant extracts modulate gut microbiota and are considered a strategy to combat obesity [3]. 

Physical activity also plays an essential role in the prevention and treatment of obesity by generating a negative energy balance and increased lipolysis in white adipose tissue (WAT). Exercise increases adiponectin, while leptin and TNFα expression are reduced [12]. Moreover, it reverts the obesity-related alterations in body weight by improving aerobic capacity, glucose metabolism, and cardiovascular and renal functions, thus helping to avoid the rebound effect and subsequent body weight regain associated with many weight-loss treatments [13]. 

Therefore, we hypothesized that a change from an obesogenic diet to a calorie-restricted diet rich in bioactive compounds, combined with a physical exercise protocol, is a valuable therapeutic strategy for the treatment of metabolic disorders derived from the development of obesity. Moreover, the dietary inclusion of a nutraceutical made from various plant extracts with high antioxidant capacities may contribute to reducing the oxidative effects of obesity and may modulate gut microbiota. The aims of this study were: (i) to design a nutraceutical with high antioxidant capacity from different seeds and plant extracts, and (ii) to study the beneficial effects of an individual or combined intervention strategy, with caloric restriction, the designed nutraceutical, and a mixed-training protocol, on different parameters of oxidative stress, inflammation, and gut dysbiosis using a mouse experimental model of diet-induced obesity (DIO) to extend our knowledge on the potential molecular mechanisms that support our experimental findings.

## 2. Materials and Methods

### 2.1. Plant Material

All seeds and plants studied were provided by the biotechnology company Cellbitec S.L. (Almería, Spain). In most cases, the plant material was seeds, except for the Sapotaceae and Araceae families in which the whole plant and the pulp of the fruit, respectively, were used. Plant material was freeze-dried and ground to reduce particle size for subsequent extraction methods.

A total of 9 families, 16 genera, and 19 species were analyzed. Only one species of the families Lamiaceae (*Ocimum basiclicum*) and Sapotaceae (*Argania spinosa*) was studied, while two were included from Amaryllidaceae (*Allium ampeloprasum* and *Allium cepa*), Araceae (*Lemna minor* and *Spirodela polyrhiza*), Brassicaceae (*Camelina sativa* and *Brassica oleracea*), Cucurbitaceae (*Cucumis sativus* and *Cucurbita pepo*), and Solanaceae (*Capsicum annuum* and *Solanum lycopersicum*). For Apiaceae and Fabaceae, three (*Eryngium maritimum*, *Eryngium bourgatii*, and *Eryngium campestre*) and four species (*Psoralea corylifolia*, *Pisum sativum*, *Cicer arietinum*, and *Vicia sativa*), respectively, were included. Most of the seed species used for the first screening were chosen because they are considered agricultural residues when they lose their germination capacity. Specifically, seeds in the families Amaryllidaceae, Brassicaceae, Cucurbitaceae, Fabaceae, and Solanaceae are common residues in south-eastern Spain, Almeria, where intensive greenhouse cultivation of these plant species is widespread and many of these seeds are discarded. Argan fruit pulp is also considered a waste product after the extraction of the oil from its kernel and the residue used in this work was provided by an organic farming facility in Andalusia (Spain).

### 2.2. Functional Extracts and Nutraceutical Formulation

The plant material was subjected to two different techniques for the extraction of bioactive compounds: an ethanolic extraction to concentrate the phenolic compounds, and an aqueous protein extraction then hydrolysis to obtain bioactive peptides. 

Ethanolic extraction. Seeds or freeze-dried material were extracted consecutively, for 30 min, with a hydroalcoholic solution of ethanol/water/HCl (50:50:0.2, *v*/*v*/*v*), on a magnetic stirrer at 4 °C and pH 2, allowing for an adequate solubility of polyphenols. After each extraction, the samples were centrifuged at 3500 rpm for 10 min and supernatant was collected while the pellet was recovered to repeat the process. Finally, all supernatants (ethanolic extract) were pooled and stored at −20 °C. Before the extraction and centrifugation processes, N_2_ was bubbled through the samples to prevent any potential oxidation as described by Kapravelou et al. [14]. 

Protein extraction and hydrolysis. Aqueous protein extraction and hydrolysis of *Spirodela polyrhiza* dried plant material and *Psoralea corylifolia* seeds were performed. *S. polyrhiza* and *P. corylifolia* were selected due to their high protein contents. The protein content of *S. polyrhiza* varies from 20 to 40% of dry weight, reaching 38.3% when it is cultured at 25 °C [15]. *P. corylifolia* seeds have a 20% of protein content [16]. For protein extraction, the protocol described by Kapravelou et al. [17] was followed. Briefly, 25 g of plant material was mixed with 100 mL of distilled water and stirred for 30 min (pH 8.8, T = 33 °C). The supernatant was collected after centrifugation at 3500 rpm for 5 min and stored at 37 °C. The extraction process was performed twice. The obtained supernatants were mixed and heated up to 47 °C for 15 min, and CaCl_2_ and MgSO_4_ 0.1 M (1:100 *v*/*v*) were added to the mixture. Hydrolysis of the extracted proteins was performed after the addition of proteases from *Bacillus licheniformis* (0.3 AU/g protein) and *Aspergillus oryzae* (100 AU/g protein) for 30 min each (pH 8.8, T = 47 °C). 

Nutraceutical formulation. Nutraceutical design was based on the combination of different extracts to provide an ideal synergy between them and potentiate their benefits for the treatment of obesity-related deleterious effects on oxidative and inflammatory markers as well as on the gut microbiome. The final selection of the ethanolic extracts for nutraceutical design was made based on the total polyphenol content responsible for their high antioxidant potential. Regarding protein hydrolyzates, the extraction yield, antioxidant capacity, and the content of bioactive peptides that would allow a semi-industrial scale production were considered. The nutraceutical was formulated by combining 10 p of ethanolic extract of *Argania spinosa* pulp with 10 p of ethanolic extract of *Camelina sativa* seeds, 40 p of protein hydrolysate from *Spirodela polyrhiza* whole plant and 40 p of protein hydrolysate from *Psoralea corylifolia* seeds. Due to differences in the freeze-drying behaviors of ethanolic extracts and protein hydrolyzates, the extracts were mixed in liquid form in the designed proportions, and the new formulation was lyophilized for its incorporation in the animals’ diet.

The required volume of each extract to meet the above formulation was calculated after the analysis of extract yields in lyophilized samples (Cryodos-50 lyophilizer, TELSTAR, Madrid, Spain). Ethanol removal from ethanolic extracts was performed by using a vacuum evaporator (ThermoSci, Waltham, MA, USA) before the freezing of the samples. 

### 2.3. In Vitro Digestion

In vitro digestion of the formulated nutraceutical was carried out using the methodology described by Porres et al. [18], with minor modifications [19]. It consisted of three phases: a first phase of gastric digestion for 2 h, followed by a 30 min pH equilibration and, finally, a 2 h intestinal digestion and absorption phase using an equilibrium dialysis process (MWCO 12,000–14,000 Da, Medicell International Ltd., London, UK). To perform the in vitro digestion process, 5 g of sample was mixed with 100 mL of 0.01 N HCl, and the pH of the reaction was adjusted to 2 with 1 N HCl. Under these conditions, 1 mL of pepsin solution (0.16 g/mL in 0.1 N HCl) was added for gastric digestion to each 20 mL digestion aliquot. After this step, dialysis bags were placed in the digestion vessels before pH compensation with 0.1 N NaHCO_3_. Then, 5 mL of a 0.1 N NaHCO_3_ solution containing pancreatin (4 mg/mL) and bile salts (25 mg/mL) was added for intestinal digestion. Once digestion was completed, the remaining contents inside (dialyzed and potentially absorbable sample) and outside (retained sample that could potentially reach the colon) the dialysis bags were collected and stored at −20 °C until further analysis was performed. Negative controls were created using the same volume of 0.01 N HCl in place of the sample. The whole process was performed in a water bath with constant agitation at 37 °C. 

### 2.4. Antioxidant Activity

The antioxidant capacity tests were carried out on the different extracts, the nutraceutical combination, and on the products obtained from the in vitro-digestibility process. Total polyphenol content was analyzed using the Folin–Ciocalteu method, with slight modifications, as previously described by Martínez et al. [20]. A standard curve of gallic acid (0–600 µg/L) was prepared. Absorbance was spectrophotometrically read at λ = 760 nm (Multiskan FC Microplate Photometer, Thermo Fisher Scientific, Waltham, MA, USA) and results were expressed as µg of gallic acid equivalent (µg GAE) per mg of extract. The ABTS total antioxidant capacity of extracts was measured according to the method of Miller et al. [21]. Six µL of evaporated ethanolic extract or a standard solution of gallic acid (0–600 µg/L) was mixed with 294 µL of ABTS (2,20-azino-bis(3-ethylbenzothiazoline-6-sulfonic acid) and incubated for 1–5 min. The optical density was measured at 620 nm (Multiskan FC, Microplate Photometer, Thermo Fisher Scientific) to evaluate the free radical scavenging capacity of the extract. The results were expressed as µg of gallic acid equivalent (GAE) per mg of extract. The complexing capacity of Fe^2+^ by the different seed extracts was assessed spectrophotometrically according to the method of Dinis et al. [22]. Reducing capacity of Fe^3+^ to Fe^2+^ conversion was analyzed according to the methods of Oyaizu [23] and Duh et al. [24]. 

The thiobarbituric acid reactive substances (TBARS) assay described by Ohkawa et al. [25] was used to measure the ability of the extract to inhibit lipid peroxidation. Rat brain homogenate was used as substrate and mixed with FeCl_3_/H_2_O_2_ to induce lipid peroxidation. After an hour of incubation at 37 °C, 1500 µL of 0.25 M HCl/15% TCA/1.34 mM DETAPAC/5% BHT, 300 µL of SDS 8.1%, and 300 µL of TBA 3% were added to the reaction and this was incubated at 75 °C for another 60 min. Finally, samples were cooled and centrifuged at 3500 rpm for 10 min. The supernatant was collected and measured at 532 nm to detect TBARS formation. The results were expressed as units of antioxidant capacity per mg of sample, calculated as previously described by Kapravelou et al. [14].

### 2.5. Mass Spectrophotometry Analysis

An Ultra Performance Liquid Chromatography (UPLC) (ACQUITY H CLASS, Waters, Milford, MA, USA) method coupled to mass spectrometry by QTOF (SYNAP G2, Waters) was employed for high-resolution mass spectrometry analysis to identify the main bioactive compounds in the extracts. Prior to mass spectrometry analysis, samples were filtered through 0.22 µm nylon disk filters (Millipore, Darmstadt, Germany). Ten microliters of the final solution was injected into the chromatograph. Analytical separation of phenolic compounds was performed on an ACQUITY HSS T33 analytical column (100 mm × 2.1 mm internal diameter, 1.8 µm) following the protocol described by Martinez et al. [17]. After chromatographic separation, high-resolution mass spectrometry analysis was carried out using negative electrospray ionization (ESIve) (Waters Corporation, Milford, MA, USA). High-purity nitrogen was used as the gas for desolvation (600 L/h) and the cone (30 L/h). Spectra were recorded over the mass/charge (*m*/*z*) range 50–1200. All compounds were identified based on their retention times (RT) and mass (MS) fragments. Based on these data, the compounds were tentatively identified using MassLynx software v4.1. 

### 2.6. In Vivo Experimental Design

#### 2.6.1. Animals

A total of 60 male C57BL/6J mice (bodyweight 18–20 g, 6 weeks old) were purchased from Charles River Laboratories Inc., Barcelona, Spain). The animals were randomly divided into six experimental groups and housed in group cages (n = 5) in a well-ventilated and thermostatically controlled room (21 ± 2 °C; Animal Experimental Unit, CIC, University of Granada, Granada, Spain). The experiments lasted for 18 weeks, allowing animals to adapt to the experimental diet, housing, and training conditions during the first week. All experiments were performed according to Directional Guides Related to Animal Housing and Care [26] and all procedures were approved by the Animal Experimentation Ethics Committee of the University of Granada, Spain (5/10/2021/148). The 3Rs principle was used to calculate the number of mice assigned to each experimental group (n = 10) [27]. 

#### 2.6.2. Experimental Design

**Figure 1:**
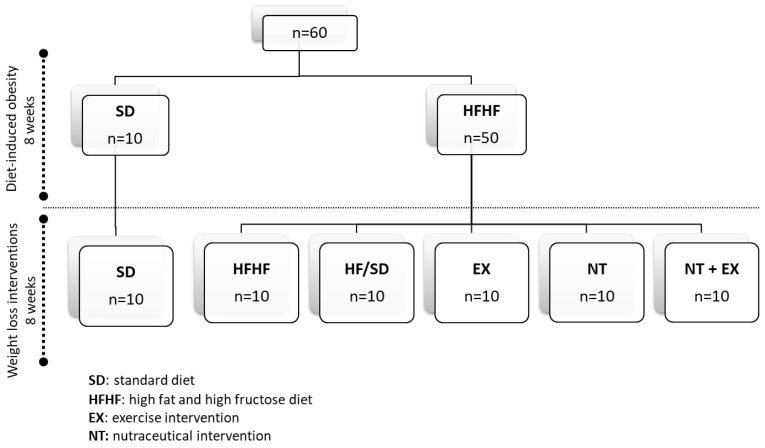
Experimental design.

The experimental groups appointed for an experimental period of 18 weeks were as follows (Figure 1): SD, a lean control group that consumed a normocaloric standard diet (Teklad Custom Diet TD110675; 3.6 kcal/g) following a pair-fed arrangement (3 g of diet/day/mouse) and unlimited access to type 2 water; HFHF, an obese control group that consumed ad libitum a high-fat diet with 20% fructose in their drinking water (Teklad Custom Diet TD06415; 4.6 kcal/g) for the entire experimental period; HF/SD, a treatment group fed a high-fat and high-fructose (HFHF) diet ad libitum during the first 9 weeks of the experimental period followed by pair-fed administration of the standard (SD) diet (3 g/mouse/day) and ad libitum administration of type 2 water during the last 9 weeks; Ex, a treatment group fed ad libitum with the HFHF diet during the first 9 weeks of the experimental period followed by pair-fed administration of the standard (SD) diet combined with a training protocol during the last 9 weeks; NT, a treatment group fed ad libitum for the first 9 weeks with the HFHF diet followed by pair-fed administration of the standard diet supplemented with 0.6% of the formulated nutraceutical (equivalent to a dose of 500 mg/kg body weight) during the last 9 weeks; and NT + Ex, a treatment group fed ad libitum with the HFHF diet during the first 9 weeks of the experimental period followed by pair-fed administration of the standard diet supplemented with 0.6% of the formulated nutraceutical combined with a training protocol for the last 9 weeks. A reversed light/dark cycle (12:12) was implemented during the entire experimental period in which all animals had unlimited access to type 2 water or fructose solution. Diet intake was recorded daily while body weight was registered individually each week at the same day and time. At the end of the experimental period, the animals were anesthetized with an intraperitoneal injection of ketamine/xylazine (75 and 10 mg/kg body weight, respectively), and the blood was collected by cardiac puncture (using heparin as an anticoagulant). An aliquot of 0.1 mL was used to assess blood parameters (KX-21 Automated Hematology Analyzer, Sysmex Corporation, Barcelona, Spain), and the rest was centrifuged at 1458 *g* for 15 min to separate plasma that was subsequently frozen in liquid nitrogen and stored at –80 °C. Several organs were removed, weighed, and visually inspected to verify the absence of macroscopic damage, and they were immediately frozen in liquid nitrogen and stored at −80 °C until they were processed for antioxidant activity assessment. The whole gastrocnemius and a piece of liver were extracted, immersed in RNA preserving solution (RNAlater, Ambion, Carlsbad, CA, USA), and stored frozen until RNA extraction. 

#### 2.6.3. Exercise Protocol

An incremental running test was performed to determine the maximum oxygen consumption (VO_2_ max) by animals as described by Yang et al. [28] with slight modifications. The test was performed using an individual treadmill (Panlab LE8708) connected to a Gas Analyzer LE 405 (Panlab, Harvard Apparatus, Holliston, MA, USA). The initial speed for the test was set at 20 cm/s, and it was progressively increased by 3 cm/s every minute up to 100 cm/s or until exhaustion of the animal was reached. For the training program, a rodent treadmill (Treadmill LE8710RTS, Panlab) was used. Mice in the Ex and NT + Ex groups were subjected to an adaptation training protocol during the 1st week, (10 min a day, slope 0, speed 12 cm/s) followed by 9 weeks of training protocol designed by our group (Table 1). The designed protocol was a high- to moderate-intensity treadmill exercise performed for 20 min a day, five days a week, during the dark cycle of the animals. Briefly, the protocol consisted of a 4 min warm-up at 20% of VO_2_ max followed by a first high-intensity cycle consisting of 3 min of a progressive increase in speed from 30 to 65% of VO_2_ max and 1 min at 65% of VO_2_ max. This was followed by four moderate-intensity sets in which a speed increase from 30 to 65% of VO_2_ max was maintained for 90 s, with additional intervals at 65% of VO_2_ max for 30 s and 1 min rest.

#### 2.6.4. Plasma Analysis

ABTS total antioxidant capacity was assayed as previously described (Section 2.4). For the quantification of TNFα, IL-6, and resistin in mouse plasma, a MILLIPLEX Kit (Mouse Metabolic Hormone Expansion Panel, MMHE-44K) was used, and absorbance was measured with a LUMINEX XYP (S/N LXY09117102). Plasma samples were completely thawed, vortexed, and centrifuged at 3000× *g* for 5 min before use. According to the MILLIPLEX Kit instructions, 200 µL of assay buffer was added to the wells. The plate was hermetically sealed and mixed for 10 min on a plate shaker at RT. The assay buffer was then removed by vigorously inverting the plate. Ten µL of assay buffer, standard solution, or sample was added to the wells and mixed with 25 µL of the premixed beads. The reaction was incubated overnight on a plate shaker at 4 °C. After that, the contents of all wells were removed, and each well was washed three times with 200 µL of wash buffer. Finally, 50 µL of detection antibodies were added to each well and the plate was incubated for 1 h, followed by the addition of 50 µL of streptavidin-phycoerythrin and incubation for 30 min. Both reactions were carried out at RT. The contents of all wells were removed and replaced by sheath fluid for reading by a LUMINEX XYP (S/N LYX 09117102). 

#### 2.6.5. Antioxidant, Detoxifying Enzyme Activity, and Lipid Peroxidation Assays in Liver

A small portion of fresh liver was homogenized in phosphate buffer 50 mM (pH 7.8) with 0.1% Triton X-100 and 1.34 mM of DETAPAC and centrifuged at 13.000× *g* for 45 min at 4 °C. The supernatant was assessed for enzyme activities and lipid peroxidation. Results were expressed related to the protein concentration of the sample, which was analyzed by the Bradford method [29]. 

Detoxifying enzymes. To assess glutathione S-transferase (GST) activity, 10 µL of 0.1 M reduced glutathione (GSH) and 10 µL of 0.1 M 1-chloro-2,4-dinitrobenzene (CDNB) were mixed with 880 µL of 0.1 M phosphate buffer (pH 6.5). This reaction mix was incubated at 37 °C for 5 min and 100 µL sample was then added to the mix. The absorbance was measured at 340 nm every minute for 5 min. GST activity was calculated as the increase in absorbance per min per mg of sample protein. To assess quinone oxidoreductase (QR) activity, the reduction of 2.6-dichloroindophenol (2.6-DCPIP) by QR was measured at 600 nm. A reaction mix containing 881.5 µL 25 mM Tris-HCl (pH 7.4), 10 µL NADH (20 mM), 5 µL FAD (10 µM), 60 µL BSA (1 mg/mL), 2.5 µL Tween (20%) and 16 µL DCIP (5 mM) was incubated at 37 °C for 5 min. Then, 975 µL of the reaction mix and 25 µL of a sample or blank (25 mM Tris-HCl) were mixed and then absorbance was measured every minute for 5 min. 

Antioxidant enzymes. Catalase activity was assayed according to Cohen et al. [30], total cellular Glutathione Peroxidase (GPx) activity was determined by the coupled assay of NADPH oxidation method described by Lawrence et al. [31] using cumene hydroperoxide as substrate. Total superoxide dismutase (SOD) activity was measured following the methodology of Ukeda et al. [32], whereas Mn-SOD activity was assessed by the same method after treating the samples with 4 mM KCN for 30 min. Cu/Zn-SOD activity resulted from the subtraction of Mn-SOD activity from the total SOD activity. 

Lipid peroxidation was analyzed in liver homogenates according to the methodology of Ohkawa et al. [25] using thiobarbituric acid reactive substances (TBARs) as a marker.

#### 2.6.6. Gene Expression Assays

Total RNA was isolated after the homogenization of plantaris muscle or liver aliquots in 1 mL Tri-Reagent (Sigma-Aldrich, St. Louis, MO, USA). RNA was solubilized in RNase-free water and treated with DNase (Applied Biosystems, Waltham, MA, USA) to remove any DNA present in the sample. A total of 100–250 ng RNA was reverse transcribed according to standard protocols using a Lifepro Thermal Cycler (Bioer Serves Life, Hangzhou, China). Quantitative RT-PCR was performed with a Quantum Studio 12 K Flex Real-Time PCR System (Applied Biosystems) using primers for genes involved in oxidative metabolism such as *sod1* (Mm01344233_g1), *sod2* (Mm01313000), *cat* (Mm00437992), and *gpx* (Mm01286848). Moreover, in the liver, genes coding detoxification pathways such as *Nqo1* (Mm00500822_g1) and *gsta2* (Mm03019257_g1), as well as markers involved in inflammatory processes, such as *Tnfα* (Mm00443258_m1), *IL-1b* (Mm00434228_m1) and *IL-6* (Mm00446190_m1) (Applied Biosystems), were also measured. The PCR master mix reaction included the first-strand cDNA template, primers, and 2X TaqMan Fast Universal PCR Master Mix, No AmpErase UNG (Applied Biosystems). ß-actin was used as an internal control. The 2 ΔΔCt method was used to analyze the data in reference to the control group. 

#### 2.6.7. Metagenomic Analysis

Cecal content was collected at the end of the experiment and used for genomic DNA (gDNA) isolation. Extraction was carried out using the QIAamp^®^ PowerFecal^®^ DNA kit following the manufacturer’s protocol for process automation with the QIAcube robot. Quantification of gDNA was performed by fluorometry (qubit). gDNA samples were analyzed by sequencing the V4 region (233 bp) of 16S ribosomal RNA (rRNA) genes using the MiSeq system (Illumina, San Diego, CA, USA). Library preparation, pooling, and miniSeq sequencing were performed at the Institute of Parasitology and Biomedicine López-Neyra (IPBLN) from the Spanish National Research Council (CSIC, Granada, Spain).

##### Bioinformatic Metagenomic Analysis

Analysis of the 16S samples was performed as recommended by Terrón-Camero et al. [33]. Briefly, following quality analysis of the two 275 bp sequences using the fastqc [34] and Multiqc software v1.14 [35], Qiime2 [36] software was used (https://qiime2.org/, accessed on 16 January 2024). This tool provides an analysis platform based on ribosomal gene databases, in this case focused on 16S from bacteria. It includes DADA2 [37] as an “external” plugin that is employed to filter, trim, and eliminate noise from the sequences, cutting both the 3’ and 5’ regions to eliminate regions with adaptor sequences as well as those showing a decrease in sequencing quality (trim-left-f and r 5, p-trunc-len-f 267, and p-trunc-len-r 275). The samples were then clustered for sample reproducibility using the EMPeror tool that identified one outlier sample, which was eliminated from this study. After the identification of the microorganisms, an abundance matrix was obtained at the family, genus, and species levels. The database used as a reference was SILVA v. 138-99 [38]. Calculation of alpha and beta diversity was also performed.

The differential abundances of the microbiomes of each experimental group were compared to the microbiome of the normocaloric group (SD) using the metagenomeSeq R package [39]. Rare species, i.e., the ones with a zero-sum of reads, were removed. To address variations in the number of reads across samples, the number of sequences was normalized using the Cumulative Sum Scale (CSS) method, using an algorithm that divides raw counts by the cumulative sum of counts to a percentile that captures the relatively invariant count distribution in the dataset. This method was used due to its higher sensitivity when compared to other normalization methods that measure taxon abundance [39]. Finally, to identify taxon changes, normalized data were subjected to differential abundance tests based on the zero-inflated Gaussian model integrated in metagenomeSeq. 

### 2.7. Statistical Analysis

Significant differences in all data sets were identified by one-way analysis of variance (ANOVA), and Tukey’s test was used to detect differences between treatment means. Student’s *t*-test was applied to test for significant differences in the in vitro digestion experiment. The effects of exercise and nutraceutical administration on hepatic antioxidant enzyme activity, plasma insulin level, and levels of inflammatory markers were also analyzed by 2 × 2 factorial ANOVA, with exercise and nutraceutical as treatments (Groups used were HF/SD, ES, NT, and NT + EX). The contribution to total variance (%) of each ANOVA component is expressed below its *p*-value. The results are given as mean values and standard error of the mean of three replicates for in vitro experiments, and of ten animals for the in vivo study. Gene expression analysis included eight animals. All analyses were performed with Statistical Package for Social Sciences (IBM SPSS for Windows^®^, version 22.0, Armonk, NY, USA). The level of significance was set at *p* < 0.05.

## 3. Results

### 3.1. Screening of Antioxidant Capacity in Different Plant Species

A total of 19 species of plants belonging to nine different families were included in this study and their total polyphenol content (TPC), as well as the antioxidant capacity of their extracts, was analyzed. The extraction yields and results of different antioxidant capacity techniques are shown in Table 2. The highest extraction yield was obtained from the ethanolic extract of *A. spinosa* pulp followed by *L. minor* (338.7 vs. 300.0) *S. polyrhiza* (263.5 ± 7.2), *C. sativa* (166.1 ± 4.9), and *P. corylifolia* (145.8 ± 2.1). Moreover, *A. spinosa* pulp extract showed the highest values for TPC as well as ABTS values. Other extracts like that of *O. basilicum* seed extract also showed a high concentration of polyphenols and a high capacity to inhibit radical ABTS formation. Species of the Fabaceae (*P. corylifolia* seed extract) and Araceae families (*L. minor* and *S. polyrhiza* extracts) showed high TPC (61.3, 44.7, and 58.8 µg GA/mg, respectively), but a lower ability to inhibit ABTS radical formation compared to other species like *Brassica oleraceae*. The highest iron-chelating capacity was found in the ethanolic extract of *L. minor*, followed by *O. basilicum LC*, *E. bourgatii*, *E. maritimum*, *C. sativa,* and *S. polyrhiza*. Finally, *O. basilicum LC* showed the greatest capacity to inhibit lipid peroxidation, followed by *E. campestre*, *S. lycopersicum*, *C. sativa*, and *S. polyrhiza*. 

### 3.2. Nutraceutical Composition

The nutraceutical was formulated based on the antioxidant capacity and extract yield results of the extracts, using the extracts with higher values in these parameters. In addition, the potential benefits of bioactive peptides on blood pressure, glucose, and lipid metabolism, as well as technological aspects related to an adequate nutraceutical preparation process, were taken into consideration. To do so, the ethanolic extracts of *Argania spinosa* pulp (10%) and *Camelina sativa* seeds (10%) and the protein hydrolysates of *Psoralea corylifolia* seeds (40%) and *Spirodela polyrhiza* whole plant (40%) were selected. Table 3 shows the antioxidant capacity of the nutraceutical formulation compared to its isolated components. The combination of the different extracts in the nutraceutical showed a greater potential for iron chelation capacity and lipid peroxidation inhibition when compared to the individual plant extracts and hydrolyzates.

**Table 2:** Extraction yields from ethanolic extracts and antioxidant activities of the different plant species analyzed.

Family	Species	Plant Material	Yield	TPC	ABTS	ICC	LPI
(n = 10)	(n = 23)		(mg/g Flour)	(µg GA eq/mg)	(µg GA eq/mg)	(CAU/mg)	(AAU/mg)
Amaryllidaceae	*A. ampeloprasum var. porrum*	Seeds	52.8 (0.9) ^cdef^	4.6 (0.5) ^a^	2.34 (0.06) ^abc^	0.67 (0.01) ^a^	0.46 (0.01) ^abc^
	*Allium cepa*		57.6 (1.1) ^defg^	6.0 (0.1) ^ab^	1.36 (0.04) ^ab^	0.92 (0.00) ^b^	0.37 (0.01) ^abc^
	*Allium cepa*		69.8 (0.4) ^fgh^	5.8 (0.1) ^ab^	1.45 (0.01) ^ab^	0.82 (0.01) ^ab^	0.22 (0.00) ^ab^
Apiaceae	*Eryngium maritimum*	Seeds	53.4 (0.6) ^cdef^	26.8 (0.4) ^e^	2.29 (0.03) ^abc^	3.74 (0.03) ^k^	0.22 (0.00) ^ab^
	*Eryngium bourgatii*		39.3 (0.4) ^abc^	35.2 (0.3) ^f^	4.93 (0.13) ^de^	4.64 (0.04) ^m^	1.14 (0.04) ^de^
	*Eryngium campestre*		38.7 (0.8) ^abc^	28.0 (0.3) ^e^	3.31 (0.08) ^bcd^	1.70 (0.04) ^fg^	2.10 (0.12) ^gh^
Araceae	*Lemna minor*	Whole plant	300.0 (3.8) ^ñ^	44.7 (0.3) ^g^	4.31 (0.06) ^cd^	5.91 (0.06) ^n^	0.83 (0.03) ^cd^
	*Spirodela polyrhiza*		263.5 (7.2) ^n^	58.8 (0.8) ^i^	7.49 (0.35) ^fg^	3.38 (0.16) ^j^	1.43 (0.06) ^ef^
Brassicaceae	*Camelina sativa*	Seeds	166.1 (4.9) ^l^	37.8 (0.9) ^f^	5.40 (0.17) ^def^	3.95 (0.08) ^k^	1.45 (0.09) ^ef^
	*B. oleracea var. Gemmifera*		129.5 (3.6) ^k^	28.6 (0.7) ^e^	11.60 (0.10) ^hi^	1.60 (0.02) ^ef^	0.48 (0.01) ^abc^
	*B. oleracea var. Gongyloides*		105.1 (1.0) ^j^	27.8 (0.3) ^e^	11.37 (0.11) ^h^	1.74 (0.01) ^fg^	0.45 (0.03) ^abc^
Cucurbitaceae	*Cucumis sativus*	Seeds	26.7 (0.4) ^a^	4.02 (0.08) ^a^	0.46 (0.01) ^a^	0.82 (0.02) ^ab^	ND
	*Cucurbita pepo*		50.3 (4.5) ^cdef^	6.66 (0.14) ^abc^	1.90 (0.05) ^ab^	2.49 (0.06) ^h^	1.53 (0.09) ^ef^
Fabaceae	*Psoralea corylifolia*	Seeds	145.8 (2.1) ^k^	61.3 (0.5) ^i^	1.82 (0.03) ^ab^	2.87 (0.01) ^i^	0.61 (0.03) ^bc^
	*Cicer arietinum*		107.9 (2.4) ^j^	2.66 (0.03) ^a^	0.33 (0.03) ^a^	1.78 (0.01) ^fg^	0.13 (0.01) ^ab^
	*Pisum sativum*		83.7 (2.5) ^hi^	6.65 (0.14) ^abc^	0.37 (0.01) ^a^	1.24 (0.01) ^cd^	0.23 (0.01) ^ab^
	*Vicia sativa*		87.9 (1.0) ^i^	23.23 (0.37) ^e^	7.12 (0.27) ^efg^	1.81 (0.03) ^fg^	0.27 (0.03) ^ab^
Lamiaceae	*O. basilicum Ligure Cert*	Seeds	46.9 (0.7) ^bcde^	62.94 (5.32) ^i^	14.99 (0.29) ^j^	4.84 (0.01) ^m^	3.25 (0.15) ^h^
	*O. basilicum Mammolo*		53.6 (1.6) ^cdef^	60.6 (3.0^) i^	8.96 (0.05) ^g^	2.30 (0.03) ^h^	1.62 (0.05) ^efg^
Sapotaceae	*Argania spinosa*	Fruit pulp	338.7 (2.8) ^o^	100.3 (0.9) ^j^	51.2 (1.6) ^l^	2.42 (0.04) ^h^	0.64 (0.01) ^bcd^
Solanaceae	*Capsicum annuum*	Seeds	30.1 (1.2) ^ab^	24.7 (8.7) ^e^	0.43 (0.05) ^a^	1.68 (0.03) ^fg^	0.57 (0.01) ^abc^
	*Capsicum annuum Jalapeño*		65.4 (1.2) ^fg^	12.3 (4.3) ^cd^	0.05 (0.01) ^a^	1.24 (0.03) ^cd^	0.20 (0.00) ^ab^
	*Solanum lycopersicum*		65.2 (2.5) ^efg^	7.29 (0.32) ^abc^	0.40 (0.01) ^a^	0.61 (0.03) ^a^	1.56 (0.03) ^ef^

TPC—total polyphenol content; ICC—Iron chelating capacity; LPI—Lipid peroxidation inhibition; GA—gallic acid; CAU—chelating activity units; AAU—antioxidant activity unit. Results (expressed per mg of extract) are means and standard error of the mean of three replicates (in parenthesis). a–o—means with different letters are significantly different (ANOVA treatment, *p* < 0.05). ND—non-detected.

**Table 3:** Antioxidant capacities of the different ethanolic extracts (EtOH) and protein hydrolysates (HP) contained in the nutraceutical formulation.

	NT	EtOH AS	EtOH CS	HP SP	HP PC
TPC (µg GA eq/mg)	40.9 (0.1) ^d^	76.4 (0.2) ^e^	36.0 (0.8) ^c^	31.9 (0.7) ^b^	23.9 (0.3) ^a^
ABTS (µg GA eq/mg)	16.1 (0.4) ^bc^	26.0 (1.2) ^d^	9.1 (0.1) ^a^	16.0 (0.6) ^b^	18.8 (0.1) ^c^
ICC (CAU/mg)	3.51 (0.04) ^d^	3.24 (0.01) ^c^	2.12 (0.01) ^b^	1.01 (0.01) ^a^	2.17 (0.01) ^b^
IRC (µg GA/mg)	25.4 (0.4) ^d^	54.2 (0.1) ^e^	23.1 (0.1) ^c^	13.0 (0.1) ^a^	15.7 (0.1) ^b^
LPI (mAAU/mg)	36.7 (3.9) ^c^	6.7 (0.2) ^a^	11.6 (0.6) ^ab^	15.7 (0.3) ^b^	11.0 (0.8) ^ab^

TPC—total polyphenol content; ICC—Iron chelating capacity; IRC—Iron reducing capacity; LPI—Lipid peroxidation inhibition (LPI); GA—gallic acid; CAU—chelating activity units; AAU—antioxidant activity units; *Argania spinosa* (AS); *Camelina sativa* (CS); *Spirodela polyrhiza* (SP); *Psoralea corylifolia* (PC). Results are means and standard error of the mean (in parenthesis) of three replicates. a–e—means with different letters are significantly different (ANOVA treatment, *p* < 0.05).

### 3.3. Mass Spectrophotometry Identification of Bioactive Compounds

The main bioactive compounds tentatively identified in the functional extracts used for the nutraceutical formulation are shown in Supplementary Table S1. A total of 51 compounds were identified, among which the most representative are flavonoids with 23 compounds ((R)-naringenin, flavodic acid, quercetin, sophoraflavanone B, barbatoflavan, volkensiflavone, dalmaisione D, dracoflavan A, lethedoside C, isoamoritin, avicularin, vitexin, xanthoangelol, hesperidin, procyanidin b2, catechin, rhoifolin, gossypin, rutin, isoquercetin, quercetin-3-a-L-arabinofuranoside, quercetin-3-O-sophoroside, and prodelphiidin B4), followed by terpenoids (apodanthoside, aquoside C, rosmanol, blumeoside C, picroside II, acevaltrate, and tingenin B) and coumarins (dihydrosamidin, scoparone, glabranin, murpanicin, and bergapten) with 7 and 5 components, respectively. In addition, four compounds were identified that belong to the isoflavonoid (hemerocallone, alpinumisoflavone, 4’–O–Methylglabridin, and mirificin) and lignan (4’-Demethylepipodohyllotoxin β-D-glucoside, hidnocarpin, glucozaluzanin C, and myrislignan) classes, three to phenylpropanoids (curcumin II, 6-Deoxyjacareubin, and methyl chlorogenate), and two to quinones (Rhein, monoacerein). Other compounds that were also identified such as vaccihein A, 2-O-caffeoylglucaric acid, and caffeic acid 3-o-glucuronide, belong to the superclasses of aromatic polyketides, glucaric acid derivatives, and glycosyl compounds, respectively. The main biological activities attributed to these compounds include antioxidant, anti-inflammatory, lipid-lowering, hypoglycemic, and anti-obesity activities. 

### 3.4. In Vitro Digestion

The results obtained from the in vitro-digestion tests are presented in Table 4. A total of 86.6% of the formulated nutraceutical was potentially absorbable after the digestion process. Furthermore, in vitro digestion did not alter the antioxidant capacity of the available nutraceutical (dialyzed), or the non-absorbable component (retained) that reaches the large intestine. In both cases, it is higher than the blank.

### 3.5. In Vivo Assay

#### 3.5.1. Food Intake and Body Weight

**Figure 2:**
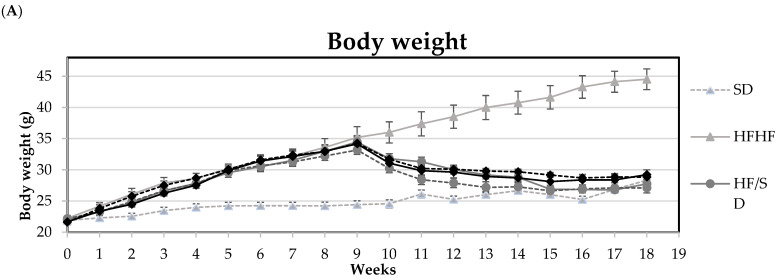

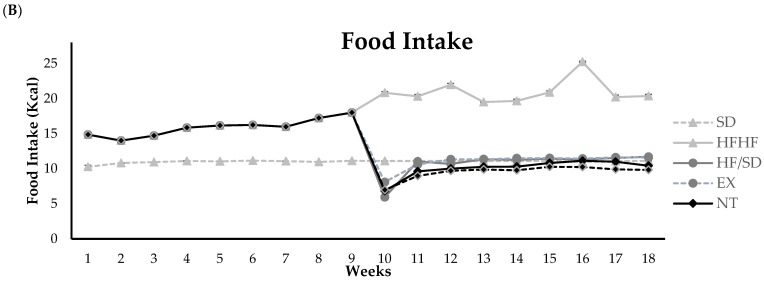
(**A**) Body weight and (**B**) Food intake of mice fed the different experimental diets. SD—Standard diet group; HFHF—high-fat, high-fructose diet group; HF/SD—nutritional intervention group of standard diet; Ex—nutritional intervention group of standard diet combined with exercise; NT—nutritional intervention group of standard diet supplemented with the nutraceutical; and NT + Ex—nutritional intervention group of standard diet supplemented with the nutraceutical combined with exercise. Results are means of 10 mice ± standard error of the mean depicted by vertical bars.

The effects of diet-induced obesity (DIO) and weight-loss interventions on the food intake and body weight of mice throughout the experimental period are presented in Figure 2. Obesity was established in the third week of the experimental period, when the weight of the animals belonging to the HFHF group was greater than twice the standard deviation of the animals in the normocaloric group SD (**1a**). In general, caloric restriction resulting from the switch from the HFHF to the SD diet after 9 weeks of obesity induction produced a significant decrease in the animals’ body weights, which was more pronounced when caloric restriction was combined with the performance of the physical exercise protocol. Regarding food intake, the HFHF group exhibited significantly lower values compared to the SD group (**1b**). During the weight-loss intervention period, experimental groups that switched to a normocaloric diet, whether or not it was combined with the training protocol (HF/SD and Ex), exhibited a similar food intake to the SD group during the whole experimental period. Animals fed the nutraceutical diet (NT and NT + Ex) had lower food intakes compared to those of the SD group, and the former parameter was even lower when the inclusion of the nutraceutical was combined with an exercise protocol.

#### 3.5.2. Tissue and Organ Weights

The weights of different organs and tissues that were collected are presented in Table 5. Obesity increased heart, spleen, abdominal, and epididymal fat weight. Conversely, caecum weight was reduced in the animals of the HFHF group and significantly increased in the NT group. The weights of liver, spleen, epididymal, and abdominal fat were reduced in response to all of the interventions applied, including caloric restriction, training protocol, and nutraceutical intake. Furthermore, the combination of nutraceutical intake and performance of the training protocol (NT + Ex group) significantly decreased kidney and heart weights after obesity development.

#### 3.5.3. Liver Parameters and Antioxidant Activity

The effects of different interventions on hepatic parameters of lipid metabolism and antioxidant enzyme activities are shown in Table 6. The HFHF group exhibited a significant accumulation of hepatic fat and, consequently, a lower percentage of water compared to the rest of experimental groups. All of the interventions assayed produced a decrease in these parameters reaching similar values to the group of animals fed the SD diet. 

Obesity induction resulted in significant increases in Cu/Zn-SOD and GST enzyme activity, while all of the interventions restored the activities of these enzymes to values similar to those of the SD group. Although the onset of obesity in our experimental model did not significantly affect Mn-SOD activity, all of the interventions tested significantly increased the activity of this enzyme. On the other hand, CAT activity was significantly higher in SD, HFHF, HF/SD, and Ex groups, whereas the combination of the nutraceutical and training protocol significantly increased GPx activity. Further statistical analysis of the obtained results comparing only the four groups of animals subjected to the different interventions showed more clearly the effects of the nutraceutical and physical exercise administration vs. the treatment group in which caloric restriction was the sole intervention (SD). In fact, physical exercise had a significant effect on the activities of all isoforms of the SOD enzyme, increasing their activities, while the opposite effect occurred with the CAT enzyme. Regarding the nutraceutical, significant effects of its consumption were observed on Mn-SOD and GPX activities that were markedly increased, while a decrease was induced in GST activity. In addition, a significant interaction was observed between the intake of the nutraceutical and the practice of physical exercise on the activity of total- and Cu/Zn-SOD activities, as both individual interventions exhibited an enhancing effect on the former parameters whereas their combination produced a decrease.

#### 3.5.4. Plasma Insulin and Inflammation Markers

The different inflammatory markers assessed in plasma are presented in Table 7. Obesity induced a significant increase in plasma insulin levels that was reverted by all of the interventions assayed, reaching values that were even lower than those reported for the SD group. However, when comparing the four experimental groups in which the interventions were carried out, the only significant effect was on nutraceutical intake. 

Plasma levels of TNFα and resistin were significantly increased by DIO, whereas exercise (Ex), dietary inclusion of the nutraceutical (NT), and their combination (NT + Ex) reverted this situation. Regarding resistin, the sole change of diet from HFHF to HF/SD proved to have a beneficial effect on this parameter. On the other hand, obesity did not affect the concentration of IL-6, although exercise (Ex), dietary inclusion of the nutraceutical (NT), and their combination (NT + Ex) significantly decreased this parameter. A deeper statistical analysis comparing only the four intervention groups showed the significant effects of exercise on plasma TNFα and IL-6, with levels that were significantly decreased vs. the HF/SD group. Likewise, a significant reducing action of the nutraceutical administration was observed in all of the plasma parameters analyzed. Furthermore, an interaction between nutraceutical intake and the training protocol affecting TNFα was observed, as both interventions individually decreased the levels of this parameter whereas a less pronounced decrease was observed when they were combined. In contrast, a synergic action was observed between both interventions for IL-6 and resistin compared to exercise alone.

#### 3.5.5. Gene Expression

The effects of obesity and different weight-loss interventions on the hepatic gene expression of inflammation markers and antioxidant enzyme transcripts are shown in Table 8. Regarding inflammatory markers, the onset of obesity affected only the gene expression of *Tnfα*, by increasing it, whereas exercise, dietary nutraceutical inclusion, and the change from an HFHF to an SD diet decreased *Tnfα* expression. In contrast, the combination of nutraceutical and exercise interventions (NT + EX) increased the gene expression of *Cat, Sod2*, *Gsta2*, and *Nqo1*. Regarding muscle gene expression, obesity did not significantly affect the antioxidant enzyme transcripts analyzed, while the interventions included in this study differentially affected these parameters. In this regard, gene expression of *Cat* was decreased by HF/SD and training protocol (EX) interventions, while it was increased in NT and NT + EX groups (all vs. HFHF group). *Gpx* gene expression was only affected by HF/SD dietary intervention and EX, with both increasing its expression (vs. HFHF group). The HF/SD intervention decreased *Sod1* and *Sod2* expression when compared to the HFHF group, while nutraceutical inclusion decreased the first and increased the second. Exercise, as well as the combination of NT + EX, increased *Sod2*. 

**Table 6:** Effects of obesity and different interventions on liver steatosis and activities of antioxidant and detoxifying enzymes.

	SD	HFHF	HF/SD	Ex	NT	NT + Ex	Ex Effect	NT Effect	NT + Ex Interaction	R^2^
Water (%)	70.4 (0.4) ^c^	66.9 (0.8) ^a^	70.5 (0.3) ^c^	69.2 (0.2) ^bc^	68.3 (0.3) ^ab^	69.9 (0.1) ^bc^	*p* = 0.295 (1.33%)	*p* = 0.002 (13.55%)	*p* < 0.001 (42.63%)	0.541
Fat (%)	3.49 (0.14) ^a^	11.63 (0.45) ^b^	3.56 (0.10) ^a^	3.93 (0.25) ^a^	4.77 (0.31) ^a^	4.08 (0.10) ^a^	*p* = 0.60 (1.08%)	*p* = 0.009 (36.48%)	*p* = 0.055 (17.00%)	0.434
CAT	7.00 (0.14) ^b^	7.01 (0.26) ^b^	7.00 (0.15) ^b^	6.10 (0.11) ^a^	6.43 (0.1) ^ab^	6.20 (0.25) ^ab^	*p* = 0.007 (16.74%)	*p* = 0.148 (4.53%)	*p* = 0.090 (6.28%)	0.213
Mn-SOD	27.3 (1.8) ^a^	25.7 (1.3) ^a^	33.8 (1.1) ^b^	35.7 (1.3) ^b^	37.5 (1.2) ^bc^	42.1 (0.9) ^c^	*p* = 0.005 (13.59%)	*p* < 0.001 (30.55%)	*p* = 0.261 (2.01%)	0.415
Cu/Zn-SOD	398.9 (11.6) ^b^	475.4 (15.0) ^c^	367.7 (7.8) ^ab^	369.7 (9.0) ^ab^	389.4 (6.6) ^ab^	346.6 (6.6) ^a^	*p* = 0.013 (14.16%)	*p* = 0.957 (0.01%)	*p* = 0.012 (14.24%)	0.223
t-SOD	428.0 (22.5) ^ab^	480.6 (20.7) ^b^	401.5 (7.9) ^a^	405.4 (9.5) ^a^	427.0 (6.7) ^ab^	388.8 (10.2) ^a^	*p* = 0.035 (10.40%)	*p* = 0.524 (0.90%)	*p* = 0.020 (12.91%)	0.177
GPx	4.65 (0.31) ^a^	5.23 (0.22) ^ab^	5.58 (0.22) ^abc^	5.61 (0.24) ^abc^	6.08 (0.28) ^bc^	6.35 (0.22) ^c^	*p* = 0.517 (1.02%)	*p* = 0.016 (15.11%)	*p* = 0.607 (0.64%)	0.096
’GST	1684.2 (67.1) ^a^	2313.7 (116.9) ^b^	2050.9 (65.5) ^b^	2105.7 (43.9) ^b^	1720.9 (81.7) ^a^	1699.6 (56.9) ^a^	*p* = 0.861 (0.07%)	*p* < 0.001 (32.03%)	*p* = 0.69 (0.34%)	0.261

SD—Standard diet group; HFHF—high-fat, high-fructose diet group; HF/SD—nutritional intervention group of standard diet; Ex—nutritional intervention group of standard diet combined with exercise; NT—nutritional intervention group of standard diet supplemented with the nutraceutical; and NT + Ex—nutritional intervention group of standard diet supplemented with the nutraceutical combined with exercise. AU—Arbitrary Units; CAT—catalase (AU/mg protein); Mn-SOD—mitochondrial superoxide dismutase (AU/mg protein); Cu/Zn-SOD—cytosolic superoxide dismutase (AU/mg protein); t-SOD—total superoxide dismutase (AU/mg protein); GPx—glutathione peroxidase nmol NADPH/min/mg protein; GST—glutathione S-transferase (AU/mg protein). Results are means and standard error of the mean (in parenthesis) of ten mice. a–c—different letters in the same row indicate statistically significant differences at a significance level of *p* < 0.05, where the letter “a” always represents the lowest mean value and the letter “c” represents the highest mean value. Data below *p*-values represent the contribution to total variance of the specific factorial ANOVA (groups HF/SD, Ex, NT, and NT + Ex).

**Table 7:** Plasma insulin and inflammation markers of mice subjected to different interventions at the end of the experimental period.

	SD	HFHF	HF/SD	Ex	NT	NT + Ex	Ex Effect	NT Effect	NT + Ex Interaction	R^2^
Insulin	600.7 (53.2) ^b^	1.096.0 (105.2) ^c^	353.8 (33.5) ^a^	315.0 (28.0) ^a^	244.3 (11.3) ^a^	204.2 (13.0) ^a^	*p* = 0.111 (3.44%)	*p* < 0.001 (26.10%)	*p* = 0.931 (0.01%)	0.258
TNFα	2.86 (0.14) ^b^	3.82 (0.24) ^c^	3.36 (0.23) ^bc^	1.21 (0.01) ^a^	1.44 (0.09) ^a^	1.69 (0.07) ^a^	*p* < 0.001 (26.02%)	*p* < 0.001 (12.89%)	*p* < 0.001 (36.47%)	0.741
IL-6	20.5 (0.4) ^b^	20.3 (0.8) ^b^	17.5 (0.8) ^b^	12.0 (1.2) ^a^	10.4 (0.5) ^a^	11.2 (1.0) ^a^	*p* < 0.001 (6.95%)	*p* = 0.013 (16.34%)	*p* = 0.002 (10.85%)	0.334
Resistin	14,098.8 (673.2) ^d^	19,344.9 (967.2) ^e^	12,070.7 (698.3) ^cd^	10,574.1 (498.2) ^bc^	7021.8 (337.6) ^a^	8539.8 (500.1) ^ab^	*p* = 0.909 (0.01%)	*p* < 0.001 (38.83%)	*p* = 0.008 (7.23%)	0.435

SD—Standard diet group; HFHF—high-fat, high-fructose diet group; HF/SD—nutritional intervention group of standard diet; Ex—nutritional intervention group of standard diet combined with exercise; NT—nutritional intervention group of standard diet supplemented with the nutraceutical; and NT + Ex—nutritional intervention group of standard diet supplemented with the nutraceutical combined with exercise. Data are expressed in pg/mL as means and standard error of the mean (in parenthesis) of eight mice. a–e—different letters in the same row indicate statistically significant differences at a significance level of *p* < 0.05, where the letter “a” always represents the lowest mean value and the letter “e” represents the highest mean value. Data below *p*-values represent the contribution to total variance of the specific ANOVA (groups HF/SD, Ex, NT, and NT + Ex).

**Table 8:** Gene expression of antioxidant, detoxifying, and anti-inflammatory transcripts in liver and plantaris muscle of mice at the end of the experimental period.

	SD	HFHF	HF/SD	EX	NT	NT + Ex
Liver gene expression						
*Cat*	1.00 (0.13) ^a^	1.11 (0.10) ^a^	1.73 (0.06) ^b^	1.84 (0.13) ^b^	2.09 (0.25) ^bc^	2.54 (0.19) ^c^
*Gpx*	1.00 (0.08) ^a^	1.04 (0.18) ^a^	1.45 (0.07) ^a^	1.22 (0.12) ^a^	1.44 (0.38) ^a^	1.25 (0.19) ^a^
*Sod1*	1.00 (0.05) ^bc^	1.19 (0.03) ^c^	0.81 (0.05) ^ab^	1.06 (0.13) ^bc^	0.62 (0.05) ^a^	1.06 (0.07) ^bc^
*Sod2*	1.00 (0.06) ^a^	1.11 (0.07) ^a^	1.40 (0.06) ^b^	1.53 (0.06) ^b^	1.07 (0.05) ^a^	1.71 (0.10) ^c^
*Nqo1*	1.00 (0.09) ^ab^	0.44 (0.06) ^a^	1.69 (0.21) ^bc^	0.61 (0.04) ^a^	0.85 (0.22) ^a^	1.85 (0.25) ^c^
*Gsta2*	1.00 (0.27) ^ab^	1.07 (0.27) ^ab^	1.39 (0.08) ^abc^	0.76 (0.02) ^a^	1.68 (0.05) ^bc^	1.87 (0.23) ^c^
*Tnfα*	1.00 (0.08) ^ab^	1.76 (0.23) ^c^	1.01 (0.11) ^ab^	0.61 (0.05) ^a^	0.86 (0.07) ^a^	1.46 (0.05) ^bc^
Plantaris muscle gene expression						
*Cat*	1.00 (0.08) ^b^	1.13 (0.08) ^b^	0.57 (0.01) ^a^	0.61 (0.04) ^a^	1.53 (0.09) ^c^	1.55 (0.07) ^c^
*Gpx*	1.00 (0.07) ^a^	1.70 (0.21) ^a^	4.04 (0.49) ^b^	3.02 (0.21) ^b^	1.41 (0.15) ^a^	1.03 (0.08) ^a^
*Sod1*	1.00 (0.05) ^cd^	0.98 (0.10) ^d^	0.64 (0.03) ^ab^	0.52 (0.04) ^a^	0.76 (0.02) ^bc^	1.16 (0.04) ^d^
*Sod2*	1.00 (0.06) ^bc^	0.92 (0.06) ^b^	0.60 (0.01) ^a^	0.85 (0.07) ^b^	1.13 (0.04) ^c^	1.44 (0.04) ^d^

SD—Standard diet group; HFHF—high-fat, high-fructose diet group; HF/SD—nutritional intervention group of standard diet; Ex—nutritional intervention group of standard diet combined with exercise; NT—nutritional intervention group of standard diet supplemented with the nutraceutical; and NT + Ex—nutritional intervention group of standard diet supplemented with the nutraceutical combined with exercise. *Cat*—Catalase; *Gpx*—Glutathione Peroxidase; *Sod1*—cytosolic superoxide dismutase [Cu-Zn]; *Sod2*—mitochondrial superoxide dismutase [Mn-SOD]; *Nqo1*—NAD(P)H: quinone oxidoreductase 1; *Gsta2*—glutathione S-transferase alpha 2; and *Tnfα*—tumor necrosis factor. Results are means and standard error of the mean (in parenthesis) of eight mice. a–d—different letters in the same row indicate statistically significant differences at a significance level of *p* < 0.05, where the letter “a” always represents the lowest mean value and the letter “d” represents the highest mean value.

#### 3.5.6. Metagenomic Analysis

**Figure 3:**
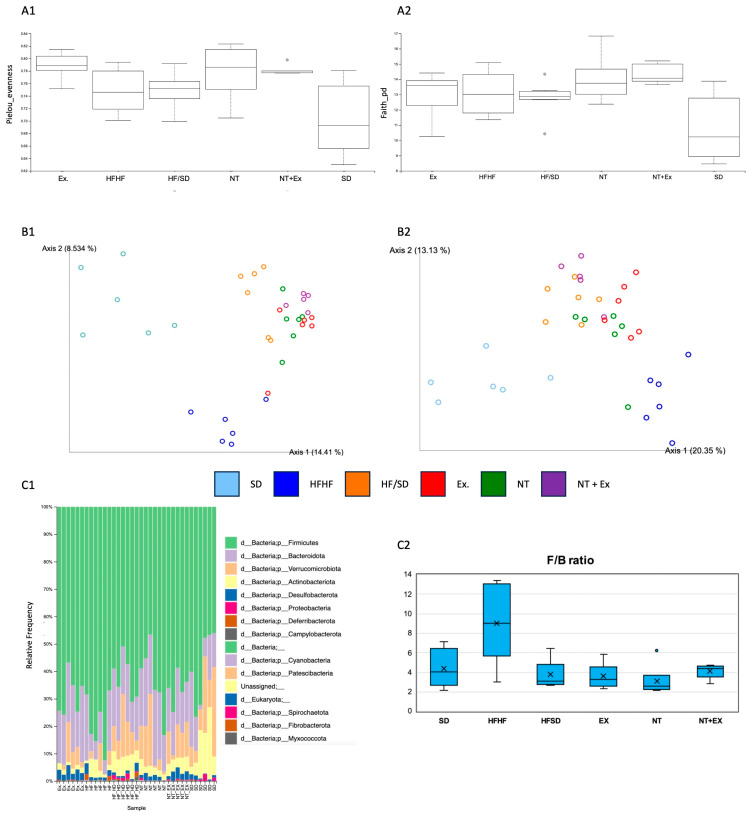
Alpha diversity expressed by Pielou index (**A1**) or faith’s phylogenetic diversity (**A2**). Beta diversity expressed by Jaccard index (**B1**) or Bray–Curtis method (**B2**). Relative abundance of the top phyla of the different experimental groups (**C1**) and Firmicutes/Bacteroidetes (F/B) ratio (**C2**). SD—Standard diet group; HFHF—high-fat, high-fructose diet group; HF/SD—nutritional intervention group of standard diet; Ex—nutritional intervention group of standard diet combined with exercise; NT—nutritional intervention group of standard diet supplemented with the nutraceutical; and NT + Ex—nutritional intervention group of standard diet supplemented with the nutraceutical combined with exercise. Results are means of 6 mice.

Metagenomic analysis of the cecum content of mice subjected to different dietary and lifestyle interventions was carried out to evaluate the impacts of DIO, exercise, and the inclusion of a nutraceutical high in bioactive compounds on gut dysbiosis. The richness or number of different species in each experimental group is shown in Figure 3. Pielou index (Figure 3(A1)) was used to calculate sample evenness, considering the number of different species and their abundances. The induction of obesity (HFHF) led to an increase in the alpha diversity index vs. the SD group, and this increase was not reversed by any of the weight-loss treatments studied. Among the latter, the highest results were attained by the Ex and NT groups, whereas the combination of nutraceutical inclusion and exercise protocol increased the alpha diversity expressed either as Pielou index (Figure 3(A1)) or faith’s phylogenetic diversity (Figure 3(A2)) when compared to SD and HF/SD groups, respectively.

Beta diversity or diversity in microbiome communities between groups is shown in Figure 3. The Jaccard index (Figure 3(B1)) evaluates the degree of similarity between groups by considering only the presence or absence of the different microorganisms. It can be observed that the distribution among individuals of each group was quite homogeneous while the relationship between individuals of different groups differed. In this regard, the SD and Ex groups showed the highest difference (further in Axis 1). Moreover, the Bray–Curtis method (Figure 3(B2)) was used to assess the degree of similarity considering the abundance of each microorganism among the experimental groups. Using this method, it can be observed that in the HFHF group abundance was decreased, a situation called dysbiosis, when compared to the SD group. The interventions implemented reversed this situation, reaching values closer to the SD group. 

The relative abundances of the phyla are shown in Figure 3. Firmicutes and Bacterioidetes were the two dominant phyla (Figure 3(C1)). The onset of obesity strongly increased the presence of bacteria belonging to the phylum of Firmicutes, and those belonging to the phyla of desulfobacterota, deferribacterota, and cyanobacteria. These populations were decreased due to the different interventions. Specifically, the phyla Firmicutes and deferribacterota showed similar values in both NT and NT + Ex groups compared to the SD group. In contrast, obesity resulted in a decrease in verrucomicrobiota, actinobacteriota, bacteroidota, and proteobacteria phyla, whereas the different interventions managed to normalize this situation, reaching similar values to the SD group except for the actinobacteriota phylum. It is worth mentioning that exercise increased the abundance of bacteriodota phylum even above the SD group. The Firmicutes/Bacteroidetes ratio (F/B) is an important marker of normal intestinal homeostasis. In the HFHF group, this ratio was significantly increased, and all of the interventions included in this study decreased the F/B ratio to even lower levels than those of the SD group. The inclusion of nutraceutical in the animals’ diet resulted in the highest decrease in this ratio (Figure 3(C2)).

Changes in relative abundance at the genus level between the groups of animals subjected to the different interventions are shown in Supplementary Figure S1. The inclusion of the nutraceutical resulted in a decrease in the abundance of the genera *Helicobacter*, *Ligilactobacillus* and *Parasutterella* while the physical exercise protocol also decreased the abundance of the former genera and increased the abundance of *Desulfovibrio* and *Clostridium methylpentosum*. However, the greatest changes in relative abundance at the genus level were found when both interventions were combined; the relative abundances of *Helicobacter*, *Ligilactobacillus,* and *Roseburia* was reduced, while those of *Desulfovibrio*, *Muribaculum*, *Prevotellaceae UGR001*, and two unidentified genera belonging to the families *Atopobiaceae* and *Ruminococcaceae*, respectively, were increased.

**Figure 4:**
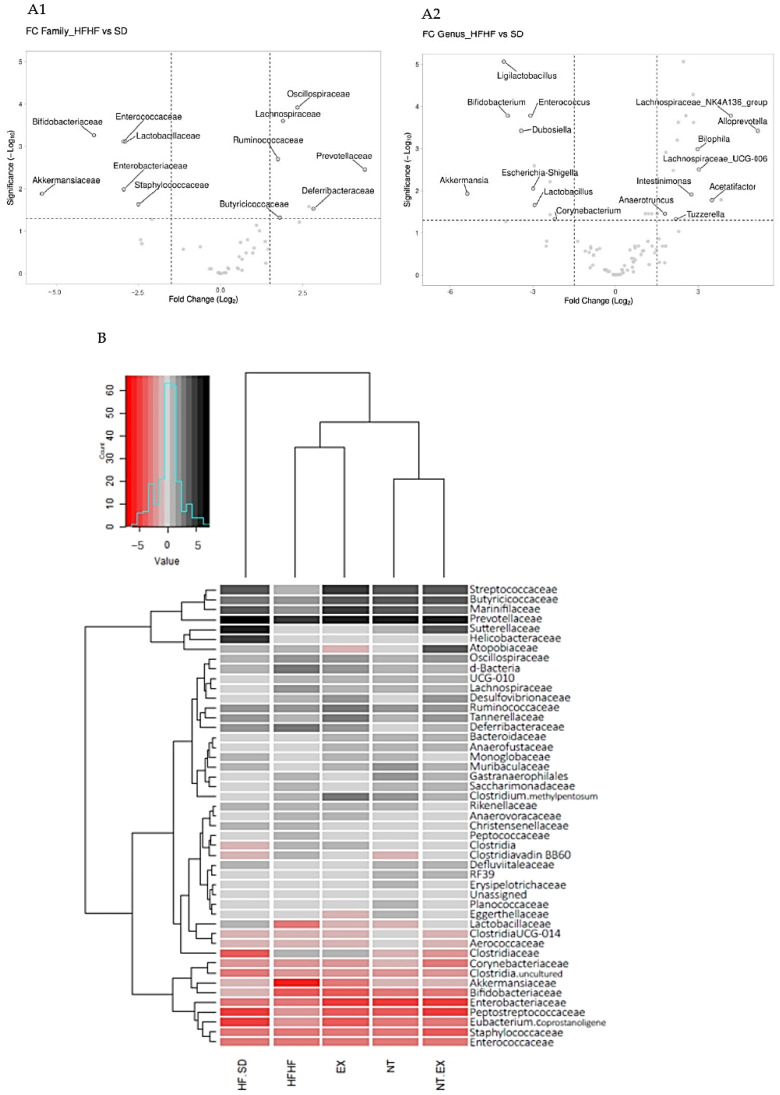
Volcano plot showing the differential abundances of the microbiome of high-fat and high-fructose diet group (HFHF) compared to the microbiome of the normocaloric group (SD) at family (**A1**) and genera (**A2**) levels. Heatmap representing the relative abundance of the top genus (**B**). Results are means of 6 mice.

Changes in fecal microorganisms between the standard diet group (SD) and the high-fat and high-fructose diet group (HFHF) are shown in Figure 4. The development of obesity caused a significant increase in *Prevotellaceae*, *Degerribacteraceae*, *Oscillospiraceae*, *Lachnospiraceae*, *Butyricicoccaceae*, and *Ruminococcaceae* families, while a decrease in *Akkermansiaceae*, *Bifidobacteriaceae*, *Enterococcaceae*, *Enterobacteriaceae*, *Lactobacillaceae*, and *Staphylococcaceae* families was found (Figure 4(A1)). At the genus level, all families, except *Butrycicoccaceae* were represented with at least one genus and showed the same trend. The most represented family was *Lachnospiraceae*, with nine overexpressed genera. The most inhibited genera were *Akkermansia*, *Ligilactobacillus,* and *Bifidobacterium* with 5.38, 3.91, and 3.42 logFC, respectively; in contrast, the most abundant genera were *Alloprevotella*, *Lachnospiraceae_NK4A136_group,* and *Lachnospiraceae_A2_group* with 5.19, 4.18, and 3.83 logFC (Figure 4(A2)).

The differences found in the microbiome of the experimental groups compared to the normocaloric group (SD), at a family level, are shown in the heatmap of Figure 4B. Obesity enhanced the abundance levels of the *Prevotelleaceae* and *Deferibacteriaceae* families, while levels of *Lactobacillaceae*, *Akkermansiaceae*, *Bifidobacteriaceae*, *Enterobacteriaceae*, *Staphylococcaceae*, and *Enterococcaceae* were decreased. The interventions included in this study increased population levels for the *Prevotellaceae* family above the levels of the HFHF group while the levels of *Deferibacteriaceae* were decreased in the NT + EX group and reached similar values to those of the SD group. The *Lactobacillaceae* family was returned to normal levels (SD group) in all experimental conditions, specifically in the group combining nutraceutical and an exercise program (NT + EX). The inclusion of nutraceutical and the change from HFHF to SD diet (NT, NT + EX groups) increased the population of *Akkermansia*. In the same way, the change in diet (HFHF to SD diet) also increased the abundance of genera in the *Bifidobacteriaceae* family to normal levels.

## 4. Discussion

Obesity is a pathology that leads to metabolic disorders such as hyperlipidemia, insulin resistance, NAFLD, impaired oxidative metabolism, and dysbiosis. There are different strategies available for reverting obesity-related alterations. Lifestyle changes like the regular practice of physical exercise and the consumption of a balanced plant-based diet, among others, may favor efficient weight loss and significantly improve overall health status. Therefore, this study aimed to test different weight-loss approaches that can reverse obesity and its associated metabolic disorders, focusing on oxidative metabolism and gut microbiome. These strategies included a change in dietary pattern to reduce the number of calories consumed, with or without a nutraceutical formulated from different plant extracts and the implementation of a physical exercise protocol. The benefits obtained from these strategies include caloric deficit but also improvements in the oxidative, inflammatory, and dysbiosis states derived from obesity.

Plants are good sources of bioactive compounds with high antioxidant capacity that can help in the treatment of obesity and associated pathologies. Thus, a screening based on the antioxidant capacity of ethanolic extracts from different plant species was carried out. Among all of the plant species studied, four of them were highlighted for their high antioxidant capacity and high extraction yield, and these were selected for the development of a nutraceutical formulation that was tested in this study. The ethanolic extract from *Argania spinosa* pulp showed the highest antioxidant capacity and extraction yield. Our previous studies have shown that argan pulp had no toxicity effects and produced beneficial effects on glycemic, lipid, and oxidative metabolism in mice after twelve weeks of oral administration [40]. However, no information is available on the physiological effects of an ethanolic extract derived from pulp, in which phenolic compounds are concentrated. Likewise, the seeds from the leguminous *Psoralea corylifolia* have been extensively studied and several compounds with antioxidant capacity such as corylisoflavone A, isopsoralen, bakuchiol, and bavachinin have been identified [41]. *P. corylifolia* seed extract was also studied as a functional material to prevent or ameliorate NAFLD in C57BL/6 mice by inhibiting lipid accumulation in the liver [42]. Regarding *Spirodella polyrhiza*, its antioxidant capacity was attributed to molecules derived from chlorogenic acid, apigenin, and luteonin [43]. On the other hand, *Camelina sativa* seed extract showed good extraction yield and great capacity to scavenge free radicals from ABTS, as well as to chelate iron. This was also demonstrated by Rahman et al. (2018) [44], who attributed this capacity to the presence of flavan-3-ol monomers as well as dimers such as procyanidin B1 and B2. In addition, the dosage of 200 mg/kg of *Camelina sativa* extract exhibited a pronounced lowering effect on parameters related to lipid metabolism in an experimental model of metabolic syndrome in rats [45]. 

The results related to in vitro-digestibility experiments showed higher antioxidant activities in the dialyzates and retentates of the nutraceutical compared to the control. Therefore, some antioxidant compounds of the former were potentially absorbable and could exert beneficial effects at the systemic level [46]. 

Under our experimental conditions, the consumption of a high-fat and high-fructose diet was able to induce obesity in an experimental model of C57BL/6J mice in agreement with what has been previously reported by Shen et al. (2023) [47]. Obesity was established in the third week of the experimental period, when the difference in mean body weights between animals fed the standard diet versus the high-fat and high-fructose diet was equal to or greater than two standard deviations [48].

Change from an HFHF to an SD diet combined with the training protocol assayed decreased animal body weight, normalizing it at the end of the experimental period to the same mean weight as that of the normocaloric group (SD). These results differ from those found by Ferrara et al. (2023) [49], who reported that a change from a hypercaloric to a hypocaloric diet failed to reverse the acquired weight to normal levels. This difference may be due to the caloric restriction induced under our experimental conditions during the intervention period, consisting of feeding 3 g of an SD diet daily to each mouse. Caloric restriction is an effective and simple strategy, which is used in weight-loss strategies as it has consistently shown beneficial effects on weight control and the prevention of metabolic dysfunctions caused by obesity [50]. Obesity is also associated with increased adiposity in other organs besides adipose tissue. In the liver, fat accumulation is of special relevance since it may lead to the development of NAFLD and affect liver functionality. As expected, our experimental model of obese mice showed higher liver weights compared to those of the experimental group fed the SD diet, as a consequence of an increased hepatic fat concentration as reported by other authors [41,48,51,52]. The inclusion of bioactive compounds in the diet [53] and the inclusion of an exercise program [54] have been reported as beneficial strategies for the reduction of lipid accumulation in the liver and other tissues. 

High-fat-diet intake is largely associated with an increase in ROS production and inflammation in metabolic tissues (liver, gastrocnemius muscle, and adipose tissue) [52], whereas intracellular antioxidant enzymes are an adequate mechanism to counteract the potential hazards associated with such alterations. Under our experimental conditions, obesity increased the levels of antioxidant (Cu/Zn-SOD) and detoxifying (GST) enzyme activity to reverse the increased obesity-related ROS production. However, other studies have shown an inverse relationship between ROS levels and antioxidant activity, revealing a reduced protective capacity of the liver in obese individuals [52,55]. The length of the experimental period is a key element for the regulation of antioxidant enzymes. It has been demonstrated that a longer experimental period of 21 weeks is effective at modulating the antioxidant capacity of the organ [48], while a shorter one (13 weeks) did not exert this action. However, in our study, higher GPx and Mn-SOD enzyme activities were found in animals fed the nutraceutical combined with an active lifestyle (NT + Ex). Furthermore, the higher hepatic gene expression levels for antioxidant (catalase and Mn-SOD) and detoxifying enzymes (GST and QR) found in the former group suggest a synergic beneficial effect between physical activity and plant-extract ingestion on the overall regulation of antioxidant mechanisms. 

Our results indicate a direct correlation between plasma levels of proinflammatory markers (IL-6 and TNFα) and hepatic mRNA levels of TNFα in obese mice. Nutraceutical supplementation and physical exercise showed a positive effect on the former, decreasing their concentrations in plasma in addition to downregulating hepatic mRNA levels. These results were also found by other authors such as De la Fuente et al. [52] and Diniz et al. [56], who reported significantly higher levels of hepatic expression of TNFα, IL-1β, and IL-6 in C57BL/6J mice fed with a high-fat and high-sugar diet. All of these parameters showed significantly lower expression when the diet was supplemented with 1.6% green tea extract [52] or when a physical exercise protocol was implemented [56]. 

High plasma resistin concentration has been related to insulin resistance and hepatic steatosis [57]. In this regard, the nutraceutical designed for our study decreased the levels of the above-mentioned marker even below the values found in the normocaloric group, therefore demonstrating its potential health benefits. This effect could be due to *Psoralea corylifolia* extract, as other authors have reported that psoralidin, a natural compound isolated from this legume, decreased resistin expression in 3T3-L1 preadipocyte cells that had been previously stimulated with adipogenic differentiation medium for 6 days [58]. 

Regarding the gut microbiome, it is well established that diet plays a key role in its composition. A diet high in sugar and/or fat induces dysbiosis in gut microbial quantity and diversity [59,60]. Although dysbiosis has been shown to cause a decrease in gut bacterial biodiversity, under our experimental conditions diet-induced obesity resulted in an increase in gut microbiome biodiversity. This result was also reported by other studies in which a higher amount of fiber was included in the HFD compared to the SD diet, and could explain the increase in gut microbial biodiversity [61]. 

The designed nutraceutical used in our study was associated with beneficial changes in gut microbiota due to its content of bioactive compounds. Polyphenol intake has been described to induce a “prebiotic-like” effect and it can modify the α-diversity of gut microbiota (increasing or decreasing it) depending on the treatment dosage, length of administration, or the type of bioactive compound administered [62]. Similarly to other authors [63], β-diversity measured by the principal-component analysis (PCA) using Bray–Curtis methodology showed that the interventions implemented in our study decreased the separation in microorganism composition found between SD and HFHF groups, making that composition closer to the SD group. In obese mice, a dysbiotic gut microbiota seems to decrease the biosynthesis of secondary bile acids that cause bacterial overgrowth [60]. 

In obese subjects and rodent experimental models, the Firmicutes phylum is increased when compared to bacteriodetes. Therefore, the Firmicutes/Bacteriodetes ratio is higher compared to that of lean subjects [64]. Firmicutes families can break complex polysaccharides to produce short-chain fatty acids (SCFAs) [65] and enhance energy harvest, thus contributing to excess lipogenesis [66]. The Bacteroidetes phylum has been suggested to be less efficient at extracting energy from food than Firmicutes, thus decreasing calorie absorption and promoting body weight loss [67]. Here, the switch from a high-fat, high-fructose diet to a standard diet decreased this ratio, whereas nutraceutical intake further reduced the values of the former index, although it did not reach significant differences compared to the HF/SD group. In addition, it improved glucose metabolism through GLP-1 release as described by other authors [68]. In this work, the higher Firmicutes/Bacteroidetes ratio found in obese animals was also related to higher plasma insulin levels. It has been described that the higher F/B ratio is associated with an increase in plasma lipopolysaccharide (LPS) levels due to an increase in intestinal permeability, causing an increase in inflammatory signaling that can lead to a decrease in IRS phosphorylation. This, in turn, can contribute to insulin resistance that results in hyperinsulinemia [68]. All of the interventions implemented in this study decreased the F/B ratio, with a parallel decrease in plasma insulin levels and a decrease in the resistance to this hormone.

At a family level, some OTUs belonging to the Firmicutes phylum, such as *Oscillospiraceae* or *Lachnospiraceae*, have been described as beneficial microorganisms that can be increased after the intake of different bioactive compounds and fiber [69]. In this study, both of these families were increased in the HFHF group, possibly due to the high content of fiber in the diet. Moreover, the nutraceutical intake, rich in bioactive compounds, as well as the exercise program, increased the abundance of these families compared to the group fed with the standard diet. *Butyricicoccaceae* abundance was upregulated in obese compared to lean mice. This family has been described as butyrate-producing bacteria [70] and was upregulated in obese mice, although the nutraceutical and exercise intervention further increased the presence of this bacteria in caecum content. Among other beneficial effects, microbiota-derived butyrate has been reported to reduce the release of pro-inflammatory cytokines into plasma [71] in a similar way to what is reported in the present study.

Obesity caused the downregulation of bacterial families belonging to *Actinobacteria* (*Bifidobacteriaceae*), *Verrucomibrobia* (*Akkermansiaceae*), *Proteobacteria* (*Enterobacteriaceae*), and *Firmicutes* (*Enterococcacae*, *Lactobacillaceae*, *Staphylococcaceae*) Phyla. The Lactobacillaceae family is increased in obese individuals [72] and decreased in lean-NAFLD. Conversely, under our experimental conditions, obesity decreased the abundance of the *Lactobacillaceae* family, and all of the interventions resulted in its increase. 

The administration of plant extracts [73], as well as of the nutraceutical in our study, represents a successful strategy to normalize the gut populations of *Akkermansiaceae* and *Bifidobacteriaceae* families that are negatively affected by obesity. Moreover, exercise modulated the diversity of gut microbiome in obese individuals and the abundance of some bacterium, mostly by increasing the abundance of *Bifidobacteriaceae* and *Akkermansiaceae* families [74]. Similarly, the training protocol applied in our study slightly increased the bacteria of the *Akkermansiaceae* family. 

## 5. Conclusions

The increasing prevalence of obesity and its related disorders demands special at-tention and efficient strategies to combat all of the health-related and social problems associated with the above-mentioned pathology. Caloric restriction is an efficient strategy for weight loss, and the dietary inclusion of a nutraceutical with high antioxidant potential can improve oxidative status and reduce inflammation markers in patients. The combination of nutraceutical administration with an exercise protocol may provide the ideal treatment synergy to be tested in the design of population-based studies to enhance the effects of caloric restriction on different metabolic and inflammatory parameters derived from the development of obesity. 

Limitations and perspective: Obesity triggers a multitude of metabolic disorders that make it a very difficult disease to assess from a clinical, nutritional, or biochemical point of view. Some of the limitations to consider in this study are the impossibility to control the daily intake of each animal or their energy consumption, as they are influenced by eating habits, individual variability, and compensatory behaviors in response to exercise. Exercise adherence or sex differences cannot be assessed in this study either. 

The development of new obesity treatments that enhance the beneficial metabolic effects of caloric restriction, novel nutraceuticals, and physical exercise is needed to treat this chronic, multifactorial, and very complex disease. The perspective of this study is to reinforce combined therapeutic strategies that help us to discover and understand molecular mechanisms and new targets for the control of these pathologies, and to extend our experimental design to future clinical studies.

## Figures and Tables

**Table 1:** Training protocol.

Stage	Time	% VO_2_ Max
Warm-up	4′	20
Exercise protocol		
1X	3′	30 → 65
	1′	65
4X	1′ 30″	30 → 65
	30″	65
	1′	rest

**Table 4:** Bioaccessibility of antioxidant capacity from nutraceutical subjected to an in vitro-digestion process.

	DIALYZED		RETAINED	
	Blank	NT	Blank	NT
Dializability (%)	-	86.6 (1.9)	-	-
TPC (µg GA eq/mg)	7.21 (0.23)	39.8 (0.7) *	8.24 (0.4)	43.7 (2.1) *
ABTS (µg GA eq/mg)	8.96 (0.23)	34.5 (0.5) *	7.05 (0.27)	21.7 (0.4) *
ICC (CAU/mg)	0.59 (0.03)	2.95 (0.14) *	0.41 (0.02)	3.87 (0.13) *
IRC (µg GA eq/mg)	0.80 (0.11)	10.1 (0.5) *	1.53 (0.16)	11.1 (0.4) *

TPC—total polyphenol content; ICC—Iron chelating capacity; IRC—Iron reducing capacity; GA: gallic acid; CAU: chelating activity units NT: nutraceutical. Results (expressed per milligram of dialyzate or retentate) are means and standard error of the mean of four replicates (in parenthesis). *—Significant differences between the blank of the test and the nutraceutical according to a Student’s *t*-test (*p* < 0.05).

**Table 5:** Weights of different organs and tissues.

	SD	HFHF	HF/SD	Ex	NT	NT + Ex
Liver	5.13 (0.06) ^bc^	5.27 (0.15) ^c^	4.70 (0.07) ^ab^	4.58 (0.11) ^a^	4.62 (0.10) ^a^	4.40 (0.12) ^a^
Kidneys	0.68 (0.01) ^ab^	0.72 (0.01) ^bc^	0.75 (0.01) ^c^	0.75 (0.01) ^c^	0.71 (0.01) ^bc^	0.67 (0.01) ^a^
Heart	0.60 (0.01) ^a^	0.66 (0.01) ^b^	0.63 (0.01) ^ab^	0.63 (0.01) ^ab^	0.64 (0.01) ^ab^	0.61 (0.01) ^a^
Spleen	0.30 (0.02) ^a^	0.40 (0.01) ^b^	0.33 (0.02) ^a^	0.30 (0.02) ^a^	0.32 (0.01) ^a^	0.30 (0.01) ^a^
Caecum	0.24 (0.01) ^bc^	0.19 (0.01) ^a^	0.24 (0.00) ^bc^	0.22 (0.01) ^ab^	0.26 (0.01) ^c^	0.27 (0.01) ^c^
Colon	0.38 (0.01) ^a^	0.50 (0.02) ^bc^	0.46 (0.01) ^b^	0.54 (0.01) ^bc^	0.48 (0.02) ^b^	0.56 (0.01) ^c^
Epididymal fat	2.44 (0.19) ^a^	8.39 (0.40) ^b^	2.69 (0.18) ^a^	2.30 (0.10) ^a^	2.72 (0.16) ^a^	2.39 (0.23) ^a^
Abdominal fat	0.62 (0.04) ^a^	2.99 (0.29) ^b^	0.68 (0.03) ^a^	0.53 (0.05) ^a^	0.83 (0.08) ^a^	0.58 (0.07) ^a^

Data are expressed as g/100 g of body weight. SD—Standard diet group; HFHF—high-fat, high-fructose diet group; HF/SD—nutritional intervention group of standard diet; Ex—nutritional intervention group of standard diet combined with exercise; NT—nutritional intervention group of standard diet supplemented with the nutraceutical; and NT + Ex—nutritional intervention group of standard diet supplemented with the nutraceutical combined with exercise. Results are means and standard error of the mean (in parenthesis) of ten mice. a–c—means within the same line with different letters are significantly different (ANOVA treatment, *p* < 0.05).

## Data Availability

Data available on request due to restrictions.

## Supplementary Materials

The following supporting information can be downloaded at: https://www.mdpi.com/article/10.3390/antiox13030274/s1, Figure S1: Changes in relative abundance at gender level, comparing the different interventions to the HF/SD group; Table S1: Identification of the main bioactive compounds in ethanolic extracts of each component used for the nutraceutical formulation.
