# Supplementary material for: A Novel Plant-Based Nutraceutical Combined with Exercise Can Revert Oxidative Status in Plasma and Liver in a Diet-Induced-Obesity Animal Model
Source: Antioxidants (Basel). 2024 Feb 23;13(3):274. doi: 10.3390/antiox13030274 (PMC10967303; doi:10.3390/antiox13030274)
Supplement: Supplementary file 1 [file antioxidants-13-00274-s001.zip › antioxidants-2842446-supplementary.pdf]

Supplementary Table S1. Identification of the main bioactive compounds in ethanolic extracts of each component used for the nutraceutical formulation.

RT	MS	COMPOUND	MF	ppm	% FIT	FRAGMENTS		
			*Argania spinosa**					
0.85	377.0927	Vaccihein A	$C_{18}H_{17}O_9$	14.3	99.17	306.0615	263.0520	180.0443
0.96	215.0361	Bergapten	$C_{12}H_7O_4$	7.9	66.72	197.0269	165.0546	159.0010
1.10	367.0999	Methyl chlorogenate	$C_{17}H_{19}O_{19}$	-9.0	91.45	293.0556	239.0853	225.0683
1.38	609.1829	Hesperidin	$C_{28}H_{33}O_{15}$	1.6	94.06	556.2178	321.0901	294.0371
2.18	371.0624	2-O-caffeoylglucaric acid	$C_{15}H_{15}O_{11}$	2.7	78.14	253.1168	239.0847	207.0574
3.26	577.1346	Procyanidin b2	$C_{30}H_{26}O_{12}$	9.0	94.94	321.1044	293.0450	275.0511
3.76	289.0748	Catechin	$C_{15}H_{13}O_6$	12.5	97.48	275.0543	239.0788	225.0721
4.00	577.1549	Rhoifolin	$C_{27}H_{29}O_{14}$	-1.4	83.53	321.0618	294.0135	275.0415
4.10	479.0853	Gossypin	$C_{21}H_{19}O_{13}$	5.6	67.63	321.0564	207.0600	179.0671
4.32	609.1522	Rutin	$C_{27}H_{30}O_{16}$	10.8	4.56	307.0088	275.0408	239.0822
4.60	463.0957	Isoquercetin	$C_{21}H_{19}O_{12}$	17.3	2.22	294.0232	179.0413	159.0064
4.87	433.0853	Quercetin 3-α-L-arabinofuranoside (Avicularin)	$C_{20}H_{17}O_{11}$	18.9	0.22	329.0309	226.9883	159.0125
5.39	625.1421	Quercetin 3-O-sophoroside	$C_{27}H_{29}O_{17}$	2.6	98.19	293.033	239.0834	226.9906
6.10	609.1296	Prodelphinidin B4	$C_{30}H_{25}O_{14}$	8.5	22.78	239.0906	225.0728	207.058
6.52	547.1502	Mirificin	$C_{26}H_{27}O_{13}$	9.1	52.18	293.0367	239.0920	225.0791
6.55	301.0388	Quercetin	$C_{15}H_9O_7$	13.3	86.01	-	-	-
			Camelina sativa					
1.05	539.1401	Blumeoside C	$C_{24}H_{27}O_{14}$	7.4	41.36	401.1378	387.1347	293.132
2.58	309.0763	6-Deoxyjacareubin	$C_{18}H_{13}O_5$	-19.7	98.91	207.008	119.0434	251.0351
2.76	511.1494	Picroside II	$C_{23}H_{27}O_{13}$	9.2	95.61	251.051	227.0063	465.1489

2.87	519.2805	Aquoside C	$C_{25}H_{43}O_{11}$	-19.3	96.93	253.1953	311.1444	441.2035
3.14	401.1084	Apodanthoside	$C_{17}H_{21}O_{11}$	18.7	82.39	207.0605	173.0217	227.0172
4.37	523.1417	Barbatoflavan	$C_{24}H_{27}O_{13}$	-5.5	0.01	227.0092	112.9958	251.048
4.96	623.1917	Volkensiflavone	$C_{36}H_{31}O_{10}$	-11.9	95.81	297.1126	227.0249	207.0365
6.43	561.1456	Dalmaisione D	$C_{27}H_{29}O_{13}$	-8.0	36	311.1276	251.0369	441.1584
7.06	793.3013	Dracoflavan A	$C_{49}H_{45}O_{10}$	-6.6	33.63	652.2723	648.2186	653.2513
7.65	479.1917	Acevaltrate	$C_{24}H_{31}O_{10}$	13.6	87.99	293.1738	251.0464	311.1239
7.89	505.1346	Lethedoside C	$C_{24}H_{25}O_{12}$	8.7	28.56	254.028	227.0302	172.9797
10.56	345.1702	Rosmanol	$C_{20}H_{25}O_5$	-10.0	20.26	265.1567	251.0438	293.1656
11.99	435.2535	Tingenin B	$C_{28}H_{35}O_4$	-6.0	43.33	353.204	325.189	266.1237
13.04	505.259	Isoamoritin	C31H37O6	7.0	82.88	397.2039	266.1328	239.0678
		Psoralea corylifolia						
2.71	355.0758	Hemerocallone	$C_{19}H_{15}O_7$	-16.9	99.81	299.0195	215.0769	191.0442
3.09	355.0722	Caffeic acid 3-o-glucuronide	$C_{15}H_{16}O_{10}$	16.1	61.17	177.0213	172.9834	149.0190
4.46	561.1682	4'-Demethylepipodohyllotoxin β-D-glucoside	$C_{27}H_{29}O_{13}$	13.2	97.83	425.1482	365.1107	253.0942
4.77	463.1098	Hidnocarpin	$C_{25}H_{19}O_9$	14.9	90.55	251.0421	172.9791	128.0853
5.57	271.0623	(R) - naringenin	$C_{15}H_{11}O_5$	6.3	98.82	149.0234	128.0166	119.0434
5.99	369.0630	Flavodic acid	$C_{19}H_{13}O_8$	5.4	98.38	267.0968	253.0851	179.0181
6.45	283.0291	Rhein	$C_{15}H_7O_6$	17.0	98.00	215.0053	205.0340	159.0141
6.59	387.1494	Dihydrosamidin	$C_{21}H_{23}O_7$	12.9	99.70	325.1483	265.1486	239.0964
6.73	301.0381	Quercetin	$C_{15}H_9O_7$	11.0	99.61			
6.79	205.0539	Scoparone	$C_{11}H_9O_4$	18.5	98.79	191.0283	164.0036	147.0019
6.97	1031.516	Funkionside F	$C_{50}H_{79}O_{22}$	9.1	80.31	653.3042	590.2572	397.1839
7.45	325.0388	Monoacerein	$C_{17}H_9O_7$	12.3	99.22	239.0745	172.9973	137.0322
7.74	337.1111	Curcumin II	$C_{20}H_{17}O_5$	10.4	99.15	128.02	119.0404	112.9984

10.6	339.1272	Sophoraflavanone B	$C_{20}H_{19}O_5$	11.8	97.07	323.1285	307.0518	173.0191
10.74	335.0958	Alpinumisoflavone	$C_{20}H_{15}O_5$	11.6	95.31	267.0569	240.0162	227.0439
11.41	323.1334	Glabranin	$C_{20}H_{19}O_4$	15.8	97.5	279.0573	253.0786	137.0235
11.79	337.1401	4' - O - Methylglabridin	$C_{21}H_{21}O_4$	-11.6	98.3	309.1794	254.0658	251.0394
12.17	321.1158	Phaseolin	$C_{20}H_{17}O_4$	9.7	98.77	279.0714	251.0397	179.0572
Spirodela polyrhiza								
3.44	433.0771	Avicularin	$C_{20}H_{17}O_{11}$	5.8	12.1	226.9891	205.0535	161.0407
4.22	431.0978	Vitexin	$C_{21}H_{19}O_{10}$	11.1	44.4	293.1036	226.9904	205.0513
6.34	407.1706	Glucozaluzanin C	$C_{21}H_{27}O_8$	17.7	91.84	343.1792	265.145	239.087
6.97	391.1909	Xanthoangelol	$C_{25}H_{27}O_4$	-14.8	99.26	265.122	173.0271	159.0364
7.92	373.1651	Myrislignan	$C_{21}H_{25}O_6$	17.7	99.99	301.135	190.0971	173.0159
15.99	303.1232	Murpanicin	$C_{17}H_{19}O_5$	-6.6	73.6	265.1399	258.0806	226.9929

TR: time retention, **MF**: molecular formula, **MS**: molecular mass. *Results previously published by Martinez et al. [42].

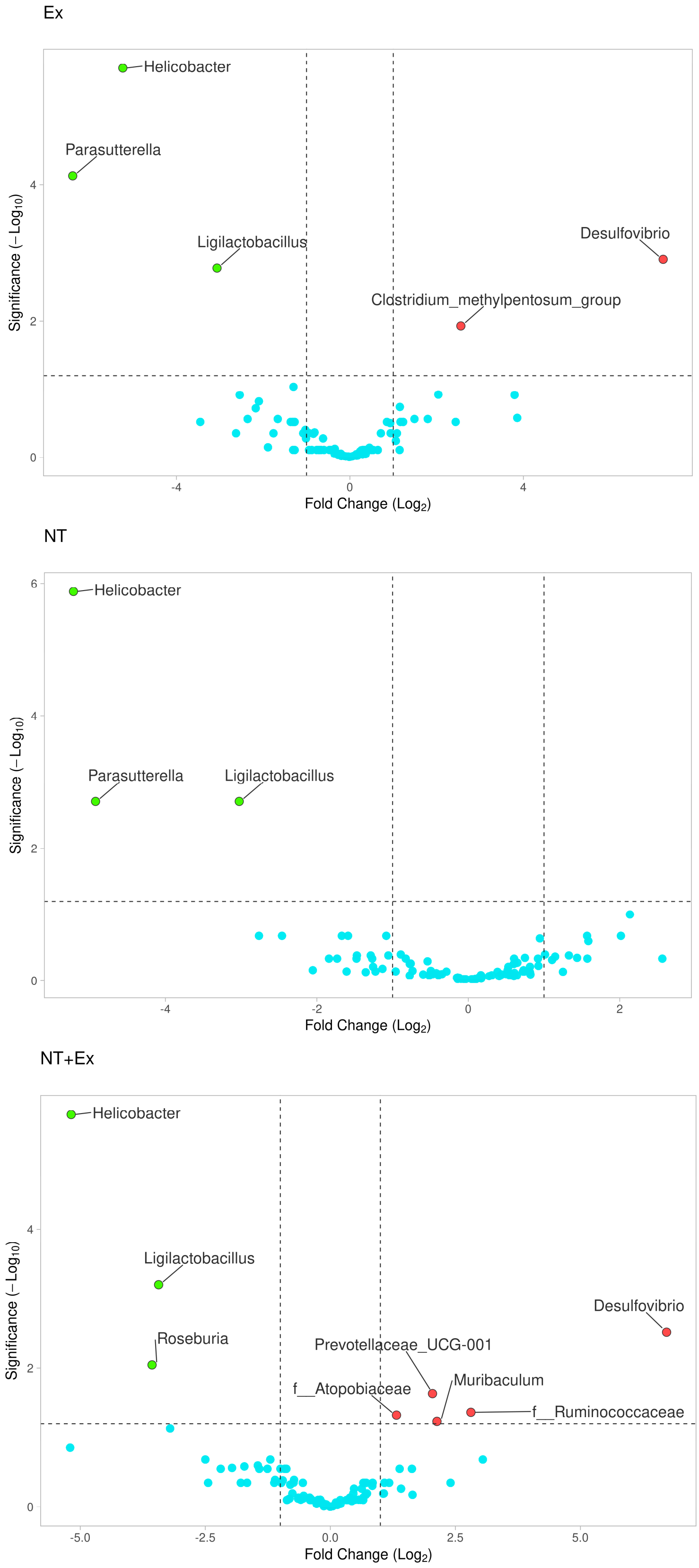

Supplementary Figure S1. Changes in relative abundance at gender level, comparing the different interventions to the JF/SD group.
